# Experimental Study on Wet Skid Resistance of Asphalt Pavements in Icy Conditions

**DOI:** 10.3390/ma12081201

**Published:** 2019-04-12

**Authors:** Boxiang Yan, Huanhuan Mao, Sai Zhong, Pengfei Zhang, Xiaoshan Zhang

**Affiliations:** 1 State Key Laboratory of Silicate Materials for Architectures, Wuhan University of Technology, Wuhan 430070, China; yanboxiang@whut.edu.cn (B.Y.); pengfeizhang@whut.edu.cn (P.Z.); zxs971206@163.com (X.Z.); 2 School of Information and Mathematics, Yangtze University, Jingzhou 434000, China

**Keywords:** asphalt pavement, skid resistance, durability, antiskid surface, image analysis

## Abstract

In this research, the durability of skid resistance during the ice melting process with temperature increasing from −5 °C to 10 °C was characterized by means of a British Pendulum Skid Tester. Four types of pavement surfaces were prepared and tested. The difference between two antiskid layers prepared with bitumen emulsion was the aggregate. The detailed angularity and form 2D index of fine aggregates used for antiskid surfaces, characterized by means of the Aggregate Image Measure System (AIMS) with micro image analysis methods, were then correlated with British Pendulum Number (BPN) values. Results indicate that skid resistance has the lowest value during the ice-melting process. The investigated antiskid layers can increase the surface friction during icy seasons. In icy conditions, the skid resistance behavior first worsens until reaches the lowest value, and then increases gradually with increasing temperature. Results from ice-melting conditions on four investigated pavement surfaces give the same temperature range where there will be lowest skid resistance. That temperature range is from 3 °C to 5 °C. A thicker ice layer will result in a lower skid resistance property and smaller “lowest BPN”.

## 1. Introduction

Asphalt pavements, considered as flexible pavements, are widely used in both road and airfield engineering. It was reported that more than 90% of highways were constructed by asphalt materials [1,2]. A durable and sustainable designed asphalt pavement would contribute a lot to economic, social and environmental aspects, as well as enhancing driving safety.

At all stages of pavement service life, the surface should have some sort of roughness to ensure sufficient friction between traffic wheels and the pavement surface [3,4]. Skid resistance is a measure of resistance of pavement surface to sliding or skidding of the vehicle [5,6,7]. The texture of pavement surface is of prime importance in providing skid resistance. It is a common fact that the lower the skid resistance, the higher the percentage of traffic accidents, especially in the wet and winter seasons.

Typically, the Locked Wheel Skid Trailer (LWST) and British Pendulum Tester (BP Tester) are the most common and accepted methods of measuring pavement skid resistance. LWST, widely used in the United States, is standardized in ASTM E 274 specifications and measures the sliding force of a locked tire on a wetting pavement surface. The BP Tester is one of the most widely used devices to determine low speed related skid resistance in the laboratory. The value obtained is called the British Pendulum Number (BPN).

However, one of the limitations of the current BP Tester is that it lacks any correction for temperatures. Previous research had concluded that BPN values are dependent on the pavement temperature [8]. Temperature influences friction properties because it changes the physical properties of tire rubber and asphalt pavement surfaces, which are both viscoelastic materials. Bazlamit reported that skid resistance of asphalt pavement decreases during seasons with warmer temperature and increases during seasons with colder temperature. Such phenomenon is related to the stiffness properties of asphalt materials and rubber that used during skid resistance tests [9].

Prof. Liang used a BP Tester to characterize the temperature effect on the measured frictional properties of a hot-mix asphalt mixture surface [10]. He concluded that an increase of temperature would result in a decrease of friction, and the effect of an HMA pavement, rubber slider and water temperatures influenced the measured frictional properties significantly. Based on the data of skid resistance of a wetted pavement, Bazlamit concluded that an approximately linear relationship can be finalized between skid resistance and temperature [8]. Table 1 summarizes the temperature ranges that have been carefully studied in literatures. Obviously, most of the related work was focused on ambient temperature or pavement surface temperature higher than 10 °C.

However, cold winter conditions will influence the micro-texture of aggregates and stiffness of tire rubber itself. Hence, research on temperature effect in colder temperature range is important as well, typically in icy conditions. In a cold winter, a better understanding of skid resistance behavior will lead to safer traffic by ensuring logical deicing techniques and skid maintenance in asphalt pavements to ensure better skid resistance [18].

Accordingly, this paper reports on the durability of skid resistance in very low temperature condition, especially in an ice-melting condition lower than 0 °C, has been developed and is discussed herein. BPN values under ice melting conditions were measured to examine the skid performance of antiskid surfaces under winter road condition. The angularity and surface texture index of the used fine aggregates were characterized by mean of Aggregate Image Measure System (AIMS) with micro image analysis methods. The detailed angularity and surface texture were then correlated with BPN values under ice-melting conditions. Conclusions from this research can be used as guidance for pavement maintenance in the winter time.

## 2. Materials and Methods

Asphalt mixture gradation of AC-16 (asphalt concrete, Panjin north asphalt co. LTD, Panjin, China) and SMA-16 (stone-matrix asphalt mixture, Panjin north asphalt co. LTD, Panjin, China) were first prepared as a traditional asphalt pavement surface layer. The void content is 4.2% for AC-16 and 4% for SMA = 16. Their skid resistance at ice-melting conditions were first tested. Another two antiskid surfaces were prepared by applying bitumen emulsion and fine aggregates onto AC and SMA surfaces. The construction steps of the antiskid surface are as follows:

Firstly, beams with 380 ± 5 mm length, 50 ± 6 mm height and 63 ± 6 mm width were cut from mixture slabs. Secondly, bitumen emulsion was uniformly sprayed onto the specimen surface with a ratio of 0.7 kg/m^2^. Table 2 presents the properties of used bitumen emulsion. Thirdly, clean and fine cubic aggregates were applied to the bitumen emulsion and let them set down into bitumen film with a ratio of 2.2 kg/m^2^. Two types of aggregates, basalt and dolerite, were used. Table 3 concludes the characteristics of the used fine aggregates. Both aggregates are alkali aggregates, so that bitumen emulsion with approximately pH of 2.0 was used.

**Figure 1:**
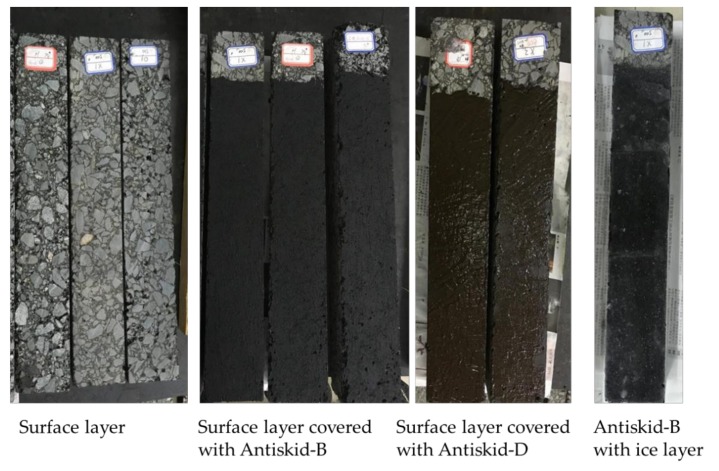
Visual feature of the investigated surfaces.

After bitumen emulsion is cured, loose aggregates were swept away and finally the antiskid layer was then obtained on the cut surfaces. Figure 1 compares the visualized features of the investigated specimens, with antiskid layer or ice layer on top. Three specimens were prepared and test for every type of antiskid surface.

### 2.1. British Pendulum Skid Test

A BP Tester was employed to measure the surface frictional properties during ice melting process in accordance with ASTM E303-93. The values measured, BPN, represent the frictional properties obtained with the apparatus. A BP Tester is a low-speed device, about 10 km/h, that measures the skid resistance related to surface micro-texture rather than macro-texture. Antiskid layers in this research used bitumen emulsion-based chip seal technology, which therefore lets the micro-texture of surface domains cause the pavement friction. Therefore, BP Tester can be used to correlate BPN during ice melting process. Table 4 shows BPN values of the investigated surfaces without ice at room temperature of 28 °C. The average BPN value of the SMA surface is 74.8, which is slightly higher than that of the AC surface. The two applied antiskid surfaces can provide better antiskid resistance, while Antiskid-B has higher increment than that of Antiskid-D.

### 2.2. Aggregates Surface Characteristics Analyses

Skid resistance is a function of different factors including the micro-texture and macro-texture of the pavement surface. Micro-texture is highly dependent on the surface characteristics of aggregates, such as angularity and surface texture [19,20]. The Aggregate Image Measure System (AIMS) is an image analysis tool, which can investigate the shape properties of both coarse and fine aggregate including angularity, sphericity and surface texture index of coarse aggregates, 2D form of fine aggregates.

**Figure 2:**
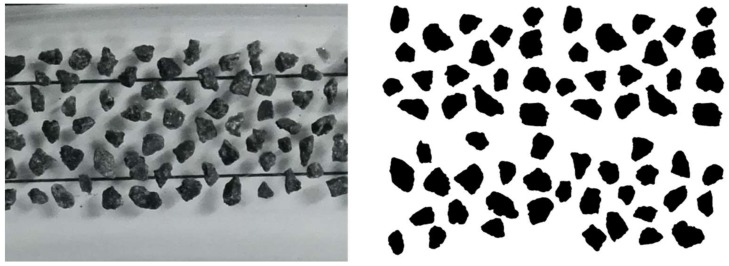
Visualized fine aggregates (left) and Aggregate Image Measure System (AIMS) image (right).

Basalt aggregate with nominal size of 0.3 to 0.6 mm, and dolerite aggregate with nominal size of 0.6 to 1.18mm, were used to prepare the antiskid surface layer. AIMS has been employed to characterize the angularity and 2D form of these two types of fine aggregate. Figure 2 presents the visualized fine aggregates and their corresponding image during AIMS analysis.

## 3. Results and Discussion

**Figure 3:**
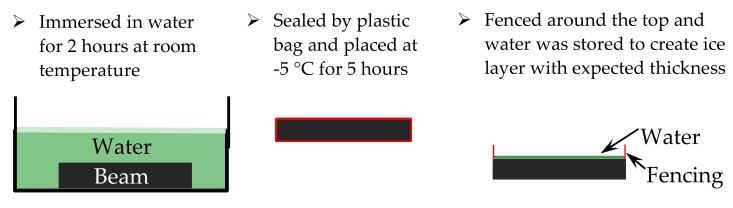
Process of prepare ice layer with certain thickness on beams.

The ice melting process has three stages, initial stage, melting stage and end stage. At the initial stage, the pavement surface is completely covered with ice, while the melting stage has both ice and water. At the end stage, the pavement is then covered with cold water and no ice present at all. Ice thickness of 1mm and 2 mm was prepared under following steps. Firstly, the beams were immersed in water for 2 h at room temperature, and then placed in the refrigerating chamber at −5 °C for 5 h with plastic bag packed. Secondly, the top side of every beam was carefully fenced and a certain amount of water was then stored to create an ice layer with the expected thickness. The first step was needed to first fill voids in the beam and hence prevent the water from leakage when it was poured onto the beam surface. This process is explained with Figure 3.

The ice-melting process was characterized by putting specimens under BP Tester at room temperature condition of 28 °C. The BPN values and surface temperatures were then continuously recorded every three minutes. In this research, the 1mm thick ice was completely melted at 7 °C, and 7.2 °C for the 2 mm thick ice layer. The surface temperature of specimens in this research was defined as the temperature of the entire specimen’s surface during the ice melting process, which means it was either temperature of the ice surface or temperature of the melted water. Therefore, the initial BPN value represents the skid resistance property of the pavement surface when it is fully covered with ice. The following BPN values illustrate the skid resistance behaves when the surface is influenced by ice and water. At the end, BPN values illustrate the surface friction characteristics under the water condition.

**Figure 4:**
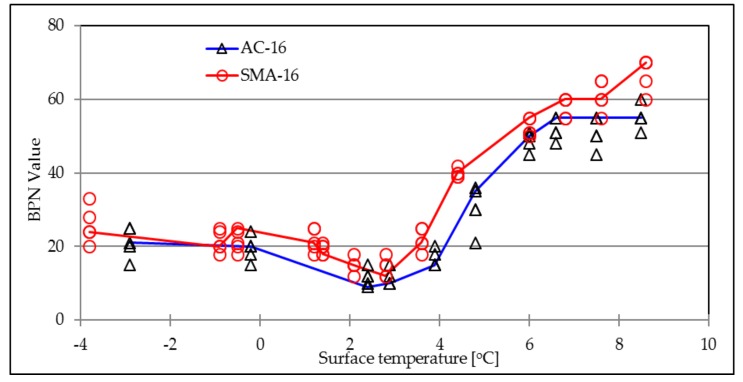
BPN values of traditional surface layers during ice-melting process.

Figure 4 compares the BPN values of AC-16 and SMA-16 under the ice-melting condition. Research data clearly illustrates that the SMA-16 surface has a higher BPN value and better skid resistance that of AC-16 surface, no matter what temperature condition and ice accumulation characteristic of the surface has. 

**Figure 5:**
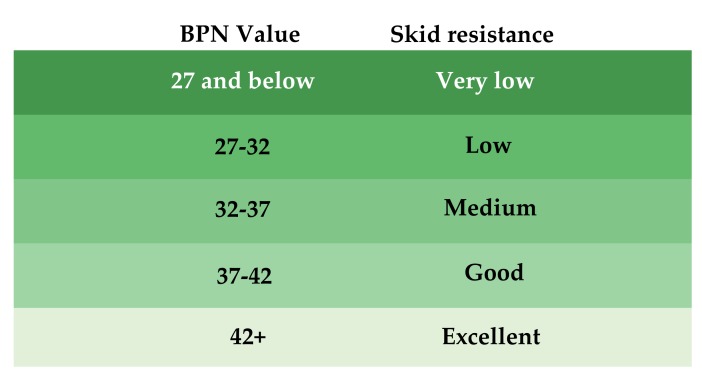
Relationship between BPN and skid resistance on pavements.

The relationship between BPN and skid resistance properties on pavements is concluded in Figure 5 [21]. It can be seen that minimum BPN of 42 must be achieved to prevent traffic from skidding accidents. This means that the investigated SMA-16 and AC-16 surface cannot provide a sufficient friction property when it is covered with ice and in the beginning of ice-melting stage. When the surface temperature increases to higher than 6 °C, both surfaces can provide BPN values higher than 42.

### 3.1. Surface Temperature Dependencies

**Figure 6:**
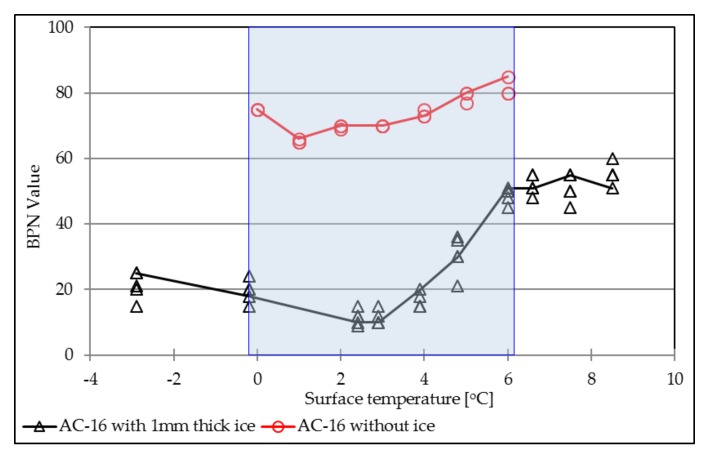
Ice effect on the BPN values.

Figure 6 indicates the ice effect on BPN values of the asphalt surface, as well as temperature influence. One millimeter ice will dramatically decrease the skid resistance of the asphalt pavement surface, the BPN values drops from 70–80 to as low as around 10. Surface temperature has the same influence contribution on both AC surface with and without the ice layer. In the range of 0 °C to 6 °C, BPN values decrease slightly and then tend to increase when the surface temperature increases.

**Figure 7:**
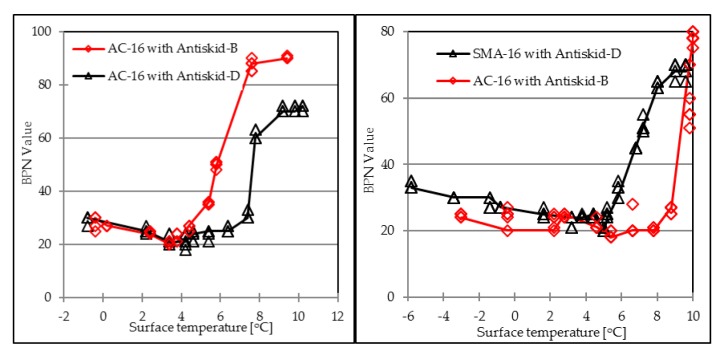
Relationship between surface temperature and BPN value.

Figure 7 presents the relationship between surface temperature and BPN values during the ice-melting process. Skid resistance at the pavement surface decreases at the very beginning of ice melting process. Initial skid resistance is relatively low until the thick ice film is worn off the top of surface by a rubber slider from the BP Tester, resulting in increasing values of BPN. Antiskid-B and Antiskid-D show the same results of BPN trends.

The ice-melting process results in a concave trend between friction and surface temperature (melting time as well). There is a temperature at which the minimum friction value at pavement surface is achieved during the ice-melting process. Table 5 concludes the lowest BPN values that occurred during ice melting at their corresponding surface temperatures. Firstly, it can clearly be seen that the investigated antiskid layers can significantly increase the surface friction properties. The lowest BPN values of AC and SMA surfaces have been enhanced from 11.6 and 14.4 to a value of around 20, respectively, when the antiskid surface has been applied. Secondly, the corresponding temperatures have been increased slightly as well, from 2.5 °C to 5 °C.

**Figure 8:**
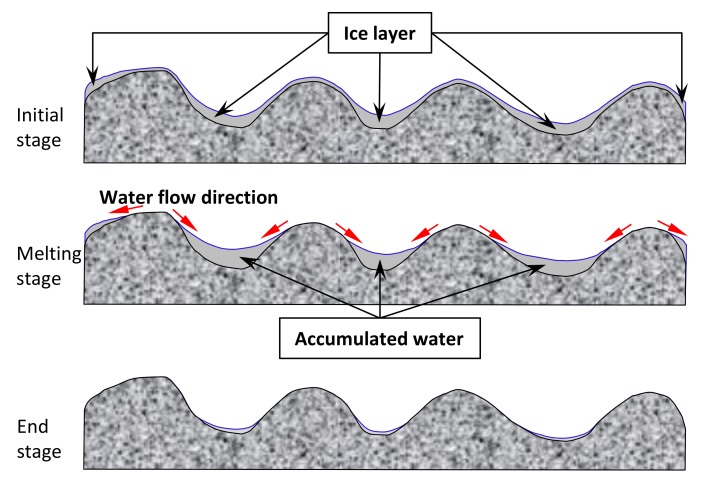
Graphic explanation on the macrotexture changes during ice melting process.

Figure 8 explains the resulting concave trend between friction and surface temperature by means of macrotexture. In the initial stage, the surface is fully covered with an ice layer. During the melting stage, water will flow from the peak to the bottom between aggregates, resulting in lower average depth between peak points and bottom points. Therefore, the lowest BPN values will be achieved in this stage. At the end stage, ice has been completely melted and water has been flowed away and evaporated as well. A lower temperature condition will result in aggregates with higher surface micro-roughness and a rubber slider of increased stiffness [22]. So the real antiskid surface has been achieved and the maximum friction value can be achieved. However, the temperature dependency in this research was concluded with an icy surface. Ice and cold water have non-negligible influence. Conducting skid resistance analysis in a cold temperature chamber could provide further valuable recommendations.

### 3.2. Aggregate and Gradation Dependencies

**Figure 9:**
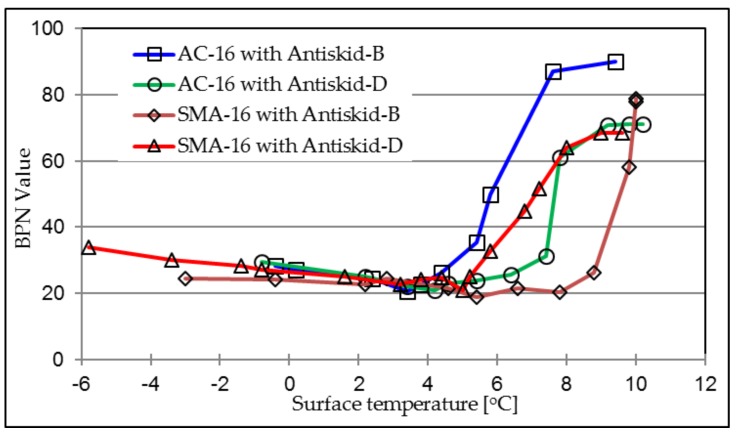
BPN values of investigated antiskid layers.

Figure 9 compares the BPN trends during the ice-melting process between different aggregate types. The ice thickness is 1 mm. The graph shows the maximum BPN values of four investigated antiskid surfaces are in agreement with the results presented in Table 4.

Antiskid-B on both AC and SMA surfaces presents the highest BPN values at the end stage, but with a far different trend during the ice-melting process. On the AC structure, friction property gets gradually enhanced when the surface temperature is higher than 4 °C, while it keeps decreasing until 8 °C and then increases significantly to the maximum value on the SMA surface. Antiskid-D presents the same BPN varying trend, as well as the maximum value at the final stage, both on AC and SMA structures.

Figure 9 illustrates that when the same surface construction method was used, basalt aggregate would provide better skid resistance than that from dolerite aggregates. In the ice-melting condition, the four curves illustrate that the surface friction will dramatically increase to the maximum BPN value within less than 6 min, at the later period in the melting stage.

**Figure 10:**
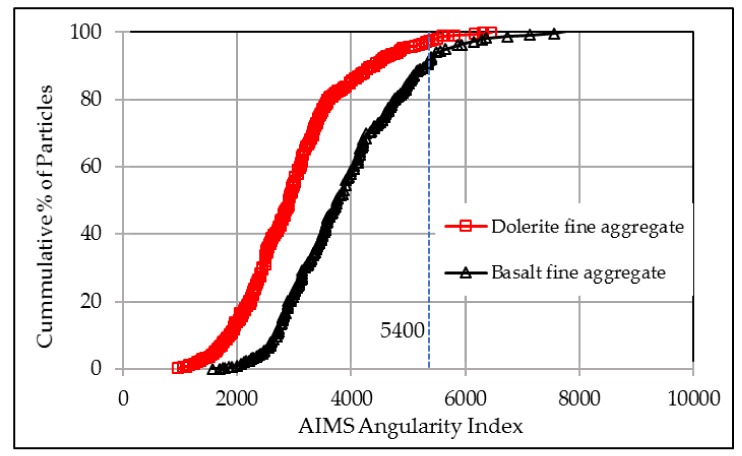
Angularity distributions of the fine aggregates used.

Angularity from AIMS analysis describes variation at the particle boundary that influences the overall shape. Higher angularity values illustrate more angular shape. A perfect circle has a small limited value of angularity, which is close to zero [23]. The definition in AIMS analysis provides a relative scale from 0 to 10,000 for angularity measurement. Figure 10 compares the angularities between basalt aggregate and dolerite aggregate.

Firstly, according to the definition of AIMS angularity, aggregates with values higher than 5400 fall into the extreme category. Figure 10 illustrates that both basalt and dolerite aggregates presented very small angularities, with less than 10% of aggregates with angularity values higher than 5400. Secondly, it can clearly be seen that basalt fine aggregate had higher angularity values than that of dolerite aggregate. According to literatures [24,25], higher angularity will result in better aggregate interlocking and higher friction coefficient, which also consistent with the BPN results discussed in Figure 9.

AIMS also defined Form2D and described the relative form from 2-dimensional images of fine aggregates, as Figure 2 shows. Form2D index is explained by Equation (1). It has a relative scale from 0 to 20, while 0 means a perfect circle.

(1) $$\mathrm{Form}2D=\sum_{\theta =0}^{\theta =360-∆\theta }\left[\frac{R_{\theta +∆\theta }-R_{\theta }}{R_{\theta }}\right],$$

where: $R_{\theta }$ is the radius of a particle at an angle of θ, ∆θ is the incremental difference in the angle.

**Figure 11:**
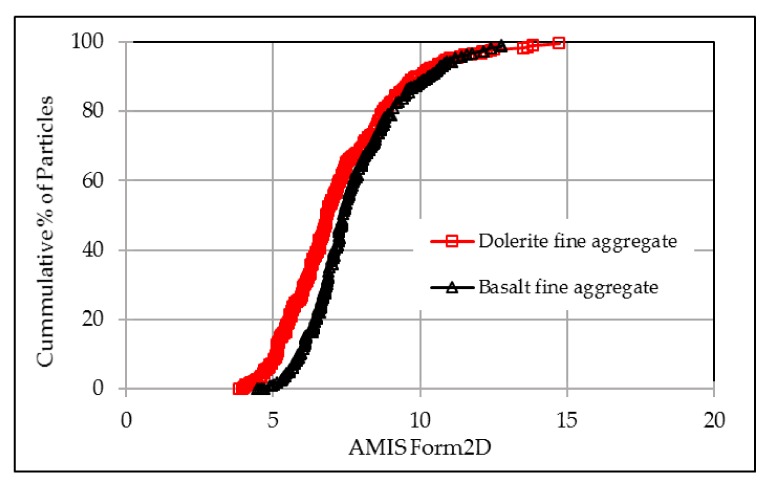
Form2D distributions of the used fine aggregates.

Figure 11 shows that basalt aggregate and dolerite aggregate have similar distributions of form2D index. This means that basalt aggregates have quite same sphericity as dolerite aggregates have, with only small difference at ranges that form2D values are smaller than 8.

### 3.3. Ice Thickness Dependencies

**Figure 12:**
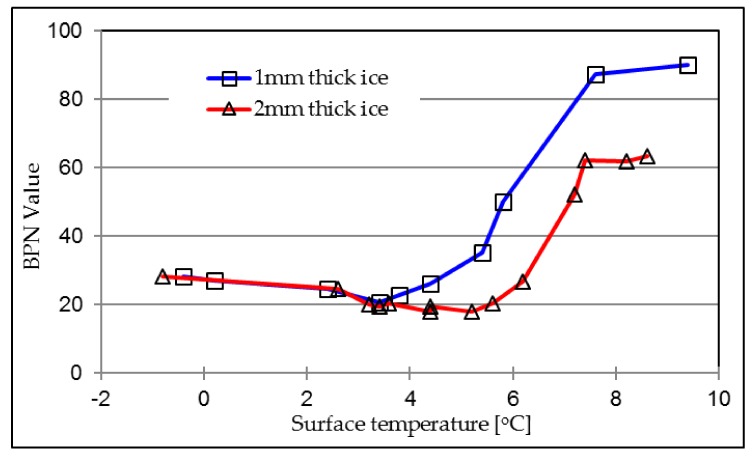
Influence of ice thickness on BPN values on asphalt concrete (AC) with antiskid-B.

Fujimoto conducted a quantitative evaluation of the relation between ice thickness on a road surface and the skid resistance property. He confirmed that the skid resistance decreases with increases in the ice film thickness [26]. This research investigates the ice thickness dependency of surface friction by comparing the BPN values during the ice-melting process between 1mm thick and 2 mm thick ice conditions. The research results are presented in Figure 12. It illustrates that thicker ice layer will result in a lower skid resistance property and smaller “lowest BPN”. The lowest BPN for the 1 mm condition is 20, while it is 18 for the 2 mm condition. Very obviously that thicker ice layer needs more time to fully melt, hence the longer melting stage.

## 4. Conclusions

The durability of skid resistance during the ice-melting process at temperature from −5 °C to 10 °C was characterized by means of a BP Tester. SMA-16, AC-16 and two types of antiskid surface layer, with 1mm thick and 2 mm thick ice layers, were studied. Based on the research results, the following conclusions can be drawn:

1. Investigated surfaces have a concave trend between friction and surface condition. Lowest BPN value occurs during the ice-melting stage, and then increases gradually with increasing temperature. Melted ice will result in water flow from peak points to gorges between aggregates, leading to lower average depth. Hence the smallest macrotexture and then lowest friction property will be presented.
2. The investigated antiskid layers can significantly increase the surface friction prope rties during icy seasons. The lowest BPN values of AC and SMA surfaces during the ice-melting process have been enhanced from 11.6 and 14.4 to a value of around 20, respectively, when an antiskid surface has been applied. Aggregate image analysis indicated that basalt fine aggregate had higher angularity values than that of dolerite aggregate, resulting in a better friction coefficient. But the lowest BPN values of the two studied antiskid layers are the same.
3. A thicker ice layer will result in a lower skid resistance property and smaller “lowest BPN”. The lowest BPN for the 1mm condition is 20, while it is 18 for the 2 mm condition.

## Figures and Tables

**Table 1:** Temperature effect on the friction behavior from literatures.

Reference	Temperature Conditions	Findings
Burchett JL, 1980 [11]	From 16.8 °C to 19 °C	Correlations between skid resistance and temperature were not good;
Elkin BL, 1980 [12]	Seasonal changes	Skid resistance was highest in the spring, dropped off noticeably during the summer, and began to recover in late fall;
Subhi MB, 2005 [8]	At 0, 10, 20, 30 and 40 °C	Skid resistance decreases with increased temperature; Linear relationship between skid resistance and temperature;
Bianchini A, 2011 [13]	From 10 °C to 35 °C	It is possible to define a reference air temperature to which friction measured at any other air temperature value can be adjusted;
Baran ED, 2011 [14]	From 10 °C to 60 °C	Temperature correction relationship for skid resistance measurements were proposed;
Robert YL, 2012 [10]	From 4.4 °C to 60 °C	Increase of temperature resulted in decrease of friction;
Scarpas A, 2013 [15]	From 0 °C to 60 °C	Effect of Pavement temperature and ambient temperature was modeled; Higher PT and AT would result in lower friction;
Hadiwardoyo SP, 2013 [16]	From 30 °C to 55 °C	Skid number values decreased with increasing temperature;
Wang DW, 2014 [17]	Surface temperature: 15–45 °C Water temperature: 10–30 °C Air temperature: 15–30 °C Tire temperature: 20–30 °C	All four of the acquired temperatures are negatively related to the friction coefficient

**Table 2:** Characteristics of bitumen emulsion for antiskid surface layer.

Properties		Values	Specifications
Appearance		Dark brown liquid	--
Density [g/cm^3^]		0.99	ASTM D6973-16
Acidity/Alkalinity		Approx. 2.0 pH	--
Viscosity at 25 °C [mPa·s]		36.7	ASTM D4402-15
Recovered binder by distillation	Weight [%]	47.5	ASTM D6997-12
	Penetration at 25 °C	62	ASTM D5-13
	Softening point	50	ASTM D36-14e1

**Table 3:** Characteristics of fine aggregates for antiskid surface layer.

Aggregate Types	Properties	Values	Specifications
Basalt	Apparent specific gravity	2.876	ASTM C128-15
	Aggregates size	0.3–0.6	
Dolerite	Apparent specific gravity	2.912	ASTM C128-15
	Aggregates size	0.6–1.18	

**Table 4:** British Pendulum Number (BPN) values of the investigated surfaces without ice.

Surface	BPNs					Average BPN	Increment
AC-16	75	70	73	70	70	71.6	--
AC16 plus Antiskid-B	80	80	81	81	80	80.4	13%
AC16 plus Antiskid-D	72	72	75	72	75	73.2	2.2%
SMA-16	76	75	73	75	75	74.8	--
SMA16 plus Antiskid-B	85	80	80	85	80	82	9.6%
SMA16 plus Antiskid-D	75	75	76	76	76	75.6	1.1%

**Table 5:** Lowest temperature and lowest BPN values.

Surface Type	Lowest BPN	Surface Temperature Where Lowest BPN Exists [°C]
AC16	11.6	2.4
AC16 plus Antiskid-B	20	3.4
AC16 plus Antiskid-D	21	4.2
SMA16	14.4	2.8
SMA16 plus Antiskid-B	18	5.4
SMA16 plus Antiskid-D	20	5
